# The Role of Inflammasomes in Chronic Oral Inflammatory Disease and Oral Cancer: A Narrative Review

**DOI:** 10.3390/dj13120609

**Published:** 2025-12-18

**Authors:** Banan Al-Natour, Issam Rasheed, Ikhlas A. El Elkarim

**Affiliations:** 1 Department of Oral Medicine and Oral Surgery, Faculty of Dentistry, Jordan University of Science and Technology, P.O. Box 3030, Irbid 22110, Jordan; ibrasheed7@just.edu.jo; 2 Wellcome-Wolfson Institute for Experimental Medicine, School of Medicine, Dentistry and Biomedical Sciences, Queen’s University Belfast, Belfast BT9 7BL, UK; i.elkarim@qub.ac.uk

**Keywords:** inflammasomes, interleukin-1beta, NF-Kappa B, chronic inflammation, pulpitis, periodontitis, carcinogenesis

## Abstract

**Background:** Chronic inflammation is a hallmark of many oral and systemic diseases and has long been recognised as a risk factor for cancer development. Central to inflammatory responses are inflammasomes—multiprotein complexes that, upon activation, trigger caspase-1–mediated release of the pro-inflammatory cytokines interleukin-1β (IL-1β) and interleukin-18 (IL-18). Their emerging contribution to chronic oral inflammatory conditions has generated interest in understanding whether persistent inflammasome activity may also influence pathways involved in oral carcinogenesis. This review summarises current evidence on the role of inflammasomes in oral inflammatory diseases and explores their potential involvement in the transition from chronic inflammation to malignant transformation. **Methods:** A narrative review of the literature was conducted by searching major scientific databases for studies investigating inflammasome activation in oral tissues, inflammatory oral diseases, and mechanisms linking chronic inflammation to oral cancer. Eligible articles included experimental studies, animal models, observational clinical research, and review papers that provided mechanistic or associative insights. Due to heterogeneity in study designs, a qualitative synthesis was performed. **Results:** Available evidence indicates that inflammasomes, particularly NLRP3 and AIM2, contribute to the pathophysiology of pulpitis, periodontitis, and several systemic conditions that affect oral health. Preclinical and observational findings also suggest potential involvement of inflammasome-related pathways in early tumorigenic processes, although these associations require further clarification. Preliminary biomarker-based studies demonstrate that inflammasome components measurable in saliva, pulpal blood, or gingival crevicular fluid may offer minimally invasive indicators of inflammatory burden and oral health status. **Conclusions:** Inflammasomes appear to play a meaningful role in oral inflammatory diseases, and growing evidence links their persistent activation to mechanisms relevant to oral carcinogenesis. However, current findings are largely associative and derived primarily from experimental and early clinical research. Additional work is needed to define precisely how inflammasomes contribute to the progression from chronic oral inflammation toward malignant change and to evaluate whether targeting inflammasome pathways offers viable therapeutic or diagnostic potential.

## 1. Introduction

Inflammation is a well-coordinated process that aims to clear infection, tissue damage and pave the way for healing and repair [1]. The typical inflammatory response is triggered upon detection of pathogenic threats and/or cellular stress via innate immune receptors termed pattern recognition receptors (PRRs) [2]. PRRs identify pathogen-associated molecular patterns (PAMPs), which are conserved molecular structures produced by microbes, and damage-associated molecular patterns (DAMPs), which are host biomolecules secreted by cells undergoing stress [3]. The activation of PAMPs initiates several downstream signalling pathways that induce the transcription and translation of many inflammatory mediators aimed at restoring tissue homeostasis [2]. Acute inflammation resolves to allow healing; however, persistent infection or impaired immune function can lead to chronic inflammation, progressive tissue damage and impaired healing. Indeed, chronic inflammation underlies numerous diseases, including cancer [4]. Emerging evidence suggests that inflammasomes play a key role in oral inflammatory conditions—such as periodontitis and pulpitis [5]—and oral cancer [6]. The evidence listed above and elsewhere [7,8,9,10] begs the question of how inflammasomes influence inflammatory oral diseases and oral cancer development.

Therefore, this review aims to integrate existing evidence on the involvement of inflammasomes in acute and chronic inflammation of oral tissues and to assess their link to oral cancer. The current evidence may clarify the mechanisms through which inflammasomes contribute to oral inflammation and carcinogenesis. Determining whether these effects arise via IL-1β secretion, NF-κB activation, or intrinsic defects in inflammasome regulation will enable more focused research in the areas that remain poorly understood and may support the development of therapeutic approaches, including boosting anti-inflammatory responses or targeting NLRP3 or AIM2 directly. Furthermore, inflammasome components or byproducts have been suggested to possess diagnostic value, as their presence in saliva or pulpal blood or gingival crevicular fluid can be measured using minimally invasive techniques. Such bio-samples could provide a practical approach to monitoring inflammatory activity and overall oral health [11,12,13].

This review was conducted as a narrative overview of the literature to highlight current knowledge on the role of inflammasomes in acute and chronic oral inflammation and their possible contribution to oral cancer. Because the aim was to explore and synthesise existing evidence rather than perform a systematic review, a non-systematic search approach was used. Most of the literature was identified through PubMed/MEDLINE, with additional articles found via Google Scholar, manual screening of reference lists, and citation tracking of key publications. Search terms included combinations of “inflammasome,” “NLRP3,” “AIM2,” “IFI16,” “dental pulp,” “pulpitis,” “periodontitis,” “chronic oral inflammation,” “interleukin-1β,” “interleukin-18,” “oral squamous cell carcinoma,” “oral cancer,” “NF-κB,” “bacterial pathogens,” and related terms.

## 2. Inflammasomes

Inflammasomes are intracellular multi-protein complexes within the cytoplasmic compartment of the cell that initiate inflammation in response to either exogenous pathogens or endogenous danger signals. Inflammasome complexes are composed of three parts: a cytosolic pattern recognition receptor (PRR), an adaptor protein (ASC), and a pro-caspase-1 enzyme [14]. Many distinct inflammasome complexes have been identified, each with a unique PRR and activation triggers. The NLR family has many receptors that assemble into inflammasomes, such as the NLRP1, the first inflammasome to be discovered [14], and the NLRP3 inflammasome, the best-studied inflammasome [15]. Of the ALR family, Absent In Melanoma (AIM2) and Interferon Inducible Protein 16 (IFI16) assemble into an inflammasome [16,17].

### 2.1. Nod-like Receptor Pyrin Domain 3 (NLRP3) Inflammasome

**Figure 1:**
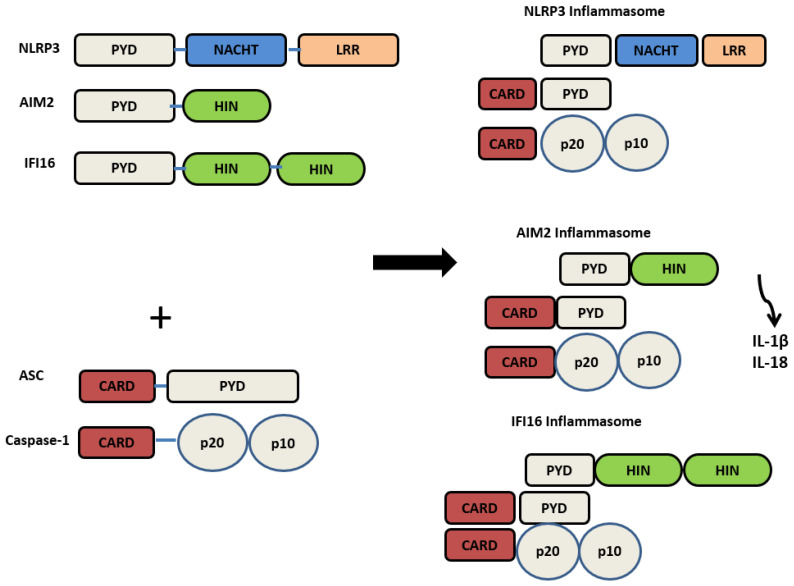
The structure of PRRs’ NLRP3, AIM2, IFI16, and their activated inflammasome counterparts.

Structurally, NLRP3 is made up of 3 domains: the (NACHT) central nucleotide-binding and oligomerisation, C-terminal (LRR) leucine-rich repeats, and N-terminal (PYD) pyrin domain. As shown in Figure 1. It has originally been perceived that the C-terminal, LRR, was involved in ligand recognition; however, studies have concluded that LRR does not function in ligand sensing. The PYD domain mediates the protein interaction with ASC (Apoptosis-associated Speck-like protein containing a CARD (Caspase activation and recruitment domain)) and subsequent downstream signalling [18].

**Figure 2:**
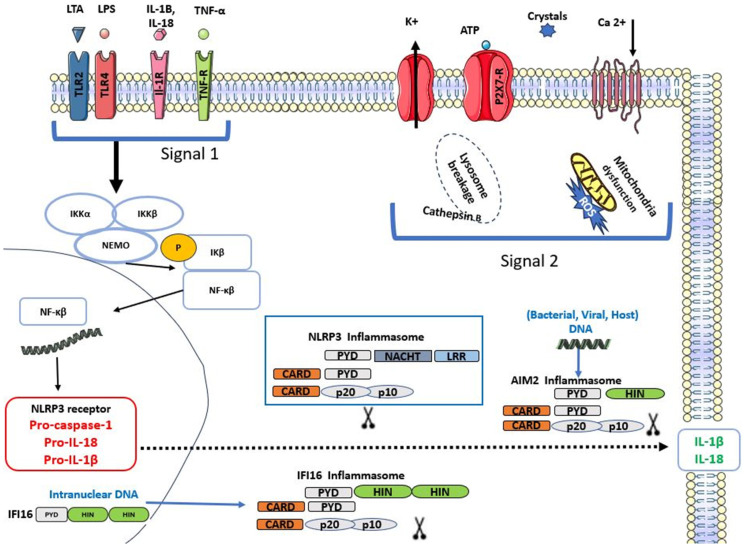
Activation of Nod-like receptor pyrin domain 3 (NLRP3), Absent In Melanoma (AIM2) and Interferon Inducible Protein 16 (IFI16) inflammasomes.

The canonical activation of the NLRP3 inflammasome requires two signals: a priming signal that induces the activation of nuclear factor kappa B (NFκB) and a second signal that induces NLRP3 oligomerisation and assembly. The first signal happens following the detection of PAMPs, DAMPs, or pro-inflammatory cytokines, which results in the transcription of NLRP3 components, interleukin-1β (IL-1β) and interleukin-18 (IL-18) via NFκB. The second signal induces NLRP3 assembly and the activation of caspase-1, which cleaves the pro-forms of IL-1β and IL-18 into their active forms and allows their release [19]. Capase-1 activation also triggers a lytic inflammatory form of cell death termed pyroptosis via Gasdermin D activation [20]. The assembly and subsequent activation of the NLRP3 inflammasome is a complex process that is not completely understood, as this inflammasome is not activated by a certain ligand. Many mechanisms have been suggested to activate the NLRP3 inflammasome, including the generation of reactive oxygen species (ROS) following DAMPs or PAMPs exposure, K+ efflux, ATP, Ca +2 flux, mitochondrial dysfunction, and release of cathepsin B via lysosomal breakage [21,22]. Figure 2 describes the activation of the NLRP3 inflammasome. The persistent exposure to inflammasome activators leads to the development of many chronic inflammatory diseases, such as gout, atherosclerosis, type 2 diabetes, cardiovascular diseases, neurodegenerative diseases, inflammatory oral disease (periodontitis and pulpitis), and cancer [5,23]. Furthermore, persistent activation of the NLRP3 inflammasome caused by genetic alterations is closely linked to various auto-inflammatory, chronic inflammatory, and metabolic diseases. For instance, familial cold autoinflammatory syndrome and Cryopyrin-associated periodic syndrome (CAPS) are caused by mutations either in NLRP3 itself or other inflammasomes [15].

### 2.2. Absent in Melanoma (AIM2) Inflammasome

AIM2 was discovered almost three decades ago in an attempt to identify novel cDNA expressed differentially in chromosome-suppressed melanoma cell lines. This study illustrated the lack of AIM2 expression in healthy melanocytes and reported a reverse tumorigenic phenotype upon AIM2 overexpression in melanocytes, which prevented tumour formation [24]. A decade later, AIM2 was identified by three separate groups to be a receptor of cytosolic dsDNA and to regulate caspase-1 via inflammasome activation [16,25,26]. It is well known that dsDNA is either confined to the nucleus or the mitochondria; hence, dsDNA in the cytosol suggests the presence of danger [3]. AIM2 senses host, viral or bacterial intracellular (dsDNA) [16]. The recognition of dsDNA induces the activation of NFκB during inflammasome activation, resulting in the maturation and release of IL-1β and IL-18 [26].

AIM2 is composed of a C-terminal Hematopoietic interferon-inducible nuclear protein with a 200 amino acid repeat (HIN200) domain that directly binds to dsDNA and an N-terminal PYD domain. AIM2 inflammasome oligomerisation is mediated by the ASC domain, which binds to the pro-caspase-1 enzyme. Due to its ability to sense dsDNA from various origins, AIM2 is involved in the pathogenesis of many diseases, including inflammatory skin disorders, inflammatory bowel diseases, chronic kidney disease, auto-inflammatory diseases, oral diseases, and cancer [27,28].

### 2.3. Interferon Inducible Protein 16 (IFI16) Inflammasomes

IFI16 recognises dsDNA in the cytosol and nucleus, mediating the induction of type 1 IFNs [29]. It contains a pyrin domain and two DNA-binding HIN domains. Similarly to AIM2, IFI16 directly binds to dsDNA via the HIN domain and responds to various sources of dsDNA [30]. IFI16 plays a crucial role in defence against viruses such as herpes simplex virus 1 and autoimmune diseases [29]. Additionally, its formation into an inflammasome seems to occur with viral DNA, as the IFI16 inflammasome assembles against the Kaposi sarcoma virus [17]. IFI16 is involved in several autoimmune diseases, such as systemic lupus erythematous, systemic sclerosis, and Sjogren’s syndrome [31], Behçet disease [32]. IFI16 was reported to be involved in oral cancer [7]. It is also involved in oral inflammatory diseases such as periodontitis [33] and pulpitis [34].

Figure 1 describes the inflammasome structure. Figure 2 demonstrates the mechanisms of inflammasome activation.

NLRP3, AIM2, and IFI16 assemble into inflammasomes by recruiting ASC and pro-caspase-1. This induces the activation of caspase-1, transforming pro-forms of IL-1β and IL-18 into their active forms and subsequent release.

The activation of Toll-like receptor (TLRs), interleukin 1-receptor (IL-1R), and tumour necrosis factor receptor (TNF-R) by their respective ligands initiates NFκB activation and translocation into the nucleus, where it transcribes pro-forms of IL-1β, IL-18, NLRP3, and pro-caspase-1. Stimulation of P2 × 7 by extracellular ATP, K^+^ efflux, and lysosome breakage in case of crystals, ROS, induces the assembly of the NLRP3 inflammasome. AIM2 inflammasome is activated upon detecting intracellular viral, host, or bacterial dsDNA. IFI16 assembles into an inflammasome following the detection of intranuclear viral dsDNA. The oligomerisation of each of these inflammasomes induces the release of IL-1β and IL-18.

## 3. The Role of Inflammasomes in Acute Pulpitis and Potential Therapeutic Targets

Cells of the dental pulp are well equipped with innate and adaptive immune responses to conquer various infectious agents [35]. The dental pulp expresses various PRRs, such as TLRs, NLRs, and ALRs [36].

TLRs are the best-characterised PRRs to date; their expression and role have been well documented in the dental pulp defence mechanisms [37,38]. In the last decade, the expression of other PRRs has been reported, such as NLRs by odontoblasts and dental pulp cells (DPCs) [37,39,40]. NLRP3 and caspase-1 are normally present in healthy dental pulp, but their expression markedly rises when the pulp becomes irreversibly inflamed [40,41]. NLRP3 activation has also been confirmed in DPCs exposed to LPS and ATP through an NFκB-dependent pathway [42,43]. Similarly, stimulation with LTA significantly increased IL-1β and IL-6 production, and this response was NLRP3-dependent, as the use of the specific NLRP3 inhibitor MCC950 reduced both cytokines [41]. More recently, NLRP3 has also been shown to drive sterile pulpal inflammation, which follows dental trauma, orthodontic movement, or operative procedures. DAMPs released from injured or necrotic pulp cells activated the NLRP3 pathway, an effect that was reduced with MCC950 or caspase-1 inhibitors [44]. Interestingly, this sterile inflammatory response also contributed to repair, as DPCs were observed migrating toward the injury site and differentiating an outcome that was dependent on IL-1β, the key byproduct of inflammasome activation. As outlined above, the NLRP3 inflammasome plays a key role in initiating acute inflammatory responses in the dental pulp, helping protect the tissue from both infectious and sterile insults. This central role suggests that NLRP3 may serve as a promising therapeutic target or even a diagnostic marker in situations where pulpal inflammation becomes excessive or dysregulated. In this context, future research should explore whether NLRP3 can be detected in pulpal blood or dentinal fluid as a screening tool for assessing the inflammatory status of the pulp. Such approaches may offer minimally invasive ways to identify dysregulated inflammation early and guide clinical decision-making. Indeed, recent studies have begun exploring dentinal fluid and pulpal blood for biomarkers capable of distinguishing different stages of pulp inflammation (reversible versus irreversible) [12,13]. Notably, IL-1β has emerged as a useful biomarker for differentiating reversible from irreversible pulpitis. Identifying these biomarkers could improve diagnostics, treatments and reduce costs.

Additionally, as mentioned briefly earlier, the NLRP3 selective inhibitor has been investigated primarily in pre-clinical models, including human dental pulp cells, ex vivo human tooth models [41], and murine studies [45]. These studies consistently show that MCC950 suppresses NLRP3 activation and reduces downstream inflammatory cytokines such as IL-1β and IL-18. However, no studies have yet evaluated MCC950 in human teeth or in clinical pulp therapy, highlighting a clear translational gap. Although initial in vitro and ex vivo findings are promising, further research using human pulp tissues, biomaterial-based delivery platforms, and eventually clinical trials is essential to determine whether MCC950 can be safely integrated into liner or capping materials to modulate inflammation and enhance pulp healing.

AIM2 expression has been identified in healthy dental pulps, with significantly higher levels observed in inflamed pulps [46,47]. Because AIM2 is activated by host- and bacteria-derived double-stranded DNA, its expression is strong in inflammatory cells and fibroblasts within inflamed pulp tissue [46,47]. Recent studies further show that AIM2 becomes activated in dental pulp cells exposed to endodontic and periodontal pathogens, including *Fusobacterium nucleatum* (*F. nucleatum*) and *Porphyromonas gingivalis* (*P. gingivalis*) [48]. AIM2 may also represent a potential therapeutic for pulpal repair. A newly identified AIM2 inhibitor, 4-sulfonic calixarenes, has been shown to reversibly bind to the HIN domain, preventing dsDNA binding and thereby reducing AIM2-dependent inflammatory signalling [49]. Interestingly, a structurally similar compound, suramin, has previously been used to inhibit ATP-mediated odontogenic differentiation of dental pulp cells through ERK inhibition [50]. This highlights an important gap and underscores the need to explore whether AIM2 inhibitors (4-sulfonic calixarenes), can be applied effectively in dental pulp tissues following the optimisation of the dosing and application to repurpose it as a pulpal medicament that can modulate the initial inflammatory response following operative procedures—when DAMPs are released from necrotic pulp cells—while still supporting DPC differentiation and tissue repair.

Together, these observations reinforce that PRRs and inflammasomes are central to initiating inflammatory responses in the dental pulp, yet these responses must be carefully regulated to support rather than hinder pulpal healing. Therapeutic strategies targeting these pathways remain in the early stages, and further research and clinical trials are needed before they can be considered for clinical use.

## 4. Chronic Periodontitis and Systemic Diseases: Is There a Role for Inflammasomes?

Chronic periodontitis is the main reason for tooth loss [51]. Oral bacterial communities, referred to as oral microbiome, exhibit balanced interaction between mutualistic and pathogenic strains, but dysbiosis elicits an inflammatory response, which results in periodontal disease [52,53]. The chronic nature of periodontitis results from a sustainable chronic inflammatory response induced by periodontal PAMPs such as LPS, LTA, unmethylated CpG-motif (CpG DNA), peptidoglycan [37,54] and tissue DAMPs released such as high mobility group box I (HMGBI), heat shock proteins (HSP) and histones released from neutrophil extracellular traps (NETs) [55,56,57]. Inflammatory cytokines such as IL-1 and TNF are the first cytokines to appear in the acute and early chronic stages of periodontitis [58]. Certainly, IL-1 is a key marker of periodontitis, with higher levels indicating more severe disease. Its actions include promoting vasodilation, chemotaxis, and promoting the secretion of tissue-degrading proteases, matrix-metalloproteinase (MMP), all of which contribute to bone resorption [59]. Nearly two decades ago, researchers measured IL-1β levels in gingival crevicular fluid (GCF). IL-1β levels correlated with probing depth (PD) and attachment loss (AL) across various stages of periodontitis. Interestingly, patients suffering from severe periodontitis showed high IL-1β regardless of pocket depth, even after treatment [60]. In recent years, saliva has emerged as a valuable tool for cytokine-based diagnostics. Salivary levels of IgA, IL-1β, and MMP-8 were higher in patients with moderate to severe periodontitis compared with healthy controls. Following periodontal treatment, these biomarker levels dropped significantly in the diseased group, while remaining unchanged in healthy individuals [61]. Indeed, salivary IL-1β proved to be a useful biomarker for identifying periodontitis and monitoring improvement after treatment, especially in non-smoking patients [62]. Additionally, salivary levels of NLRP3, ASC, and IL-1β were higher in chronic or aggressive periodontitis patients than in healthy patients; however, levels of caspase-1 were not altered [63]. Interestingly, when researchers looked at gingival tissues rather than saliva, they found higher expression of caspase-1, NLRP3, ASC and AIM2 in patients with chronic periodontitis [64]. These consistent increases across both saliva and tissue highlight the value of saliva-based inflammasome markers, particularly because saliva collection is non-invasive and far more practical than obtaining gingival biopsies. Therefore, measuring salivary NLRP3 and IL-1β may help identify the presence and severity of chronic or aggressive periodontitis, suggesting potential value for both prevention and treatment. However, unlike IL-1β, which has been examined in several studies [65]. There is still a lack of long-term research examining whether NLRP3 levels decrease after periodontal therapy.

To understand why IL-1β is so strongly expressed in periodontitis, it is also necessary to look at the bacteria that stimulate its release. Among the bacterial strains present in chronic periodontitis, *Prevotella. intermedia* (*P. intermedia*) and *F. nucleatum* are the most abundant strains in subgingival plaque samples [66]. Keystone pathogens such as *P. gingivalis*, *Aggregatibacter*. *Actinomycetemcom* + *itans* (*A. actinomycetemcomitans*), *P. intermedia*, and *T. denticola* are associated with a strong, destructive inflammatory response resulting in severe periodontitis with *P. gingivalis* as the main initiator [67,68]. The inflammatory response mediated by these keystone pathogens is instigated via PRRs and inflammasome activation [69]. *P. gingivalis*, *A. actinomycetemcomitans*, and *F. nucleatum* induce activation of NLRP3 and AIM2 inflammasomes. Furthermore, several in vitro and in vivo investigations have examined the expression of *NLRP3* and *AIM2* inflammasomes in periodontal tissues, as summarised in the following section. The expression of *NLRP3*, *NLRP2*, *IL-1β*, and *IL-18* in gingival tissues from patients with gingivitis, chronic periodontitis, and aggressive periodontitis was upregulated, with positive correlations observed between NLRP3 and its downstream products. Interestingly, *ASC* expression did not show any significant changes [70]. In line with these findings, a recent study also found that *NLRP3* was overexpressed and *NLRP2* levels were higher in patients with gingivitis and periodontitis. Their levels were closely linked with clinical signs of disease, including probing depth, attachment loss, and plaque and gingival indices [71].

In vitro, immune cells exposed to *P. gingivalis* showed an increase in *NLRP3* expression in a dose-dependent manner, in contrast to *NLRP2* and ASC [70]. Furthermore, exposure of gingival fibroblasts to supragingival biofilm promoted the expression of several inflammasome components, including *AIM2*, *caspase-1*, *ASC*, *IL-1β*, and *IL-18*, although *NLRP3* did not show the same response. In contrast, *P. gingivalis*, a key organism in subgingival biofilms, was found to downregulate both *AIM2* and *NLRP3* in gingival fibroblasts [72]. As reported in previous studies, this suppression may represent an immune-evasion strategy that *P. gingivalis* uses to persist within periodontal tissues, sustain inflammation, and contribute to ongoing tissue destruction [73]. In chronic apical periodontitis, the presence of major periodontal pathogens such as *P. endodontalis*, *F. nucleatum*, and *P. gingivalis* has also been shown to correlate with increased expression of *NLRP3* and *AIM2* in periapical lesions, suggesting that the inflammatory responses in these lesions are largely mediated through activation of these inflammasomes [70,74,75].

Differential expression of *AIM2* across studies has been noted. This differential expression of *AIM2* mainly reflects how and where it has been measured. Studies that measured *AIM2* directly in gingival biopsies found higher expression in chronically inflamed, immune-cell–rich areas [74]. Meanwhile, the *P. gingivalis* in vitro infection study used isolated cell types under acute conditions, where *AIM2* was either activated or suppressed depending on the cell type and bacterial load [64]. In a more recent study, a decrease in *AIM2* for chronic periodontitis patients was reported, with an increasing trend in the expression of caspase-1 between chronic periodontitis, aggressive periodontitis groups, and healthy subjects [71]. The overall regulatory transcripts were measured in mixed gingival tissue; this may have masked changes in individual genes like *AIM2*.

The differential expression of *AIM2* across studies mainly reflects how and where *AIM2* was measured. These differences in tissue sources, methods, and experimental settings explain the variation in *AIM2* results across the literature.

Genetic studies have also highlighted the importance of inflammasome regulation in periodontal disease. Patients with polymorphisms in the *AIM2* and *PYCARD* genes reported a susceptibility to periodontitis. As these patients exhibited elevated serum levels of IL-18 and Gasdermin D, and clinical periodontal parameters, which impacted the severity of periodontitis [33,76]. Collectively, these findings reinforce the central role of inflammasomes in the progression and development of periodontal disease. However, there is a clear need to investigate whether inflammasome blockers or inhibitors can be tested in vitro on gingival fibroblasts before progressing to localised therapeutic formulations that could be applied within periodontal pockets. It is well established that periodontitis is linked to chronic systemic inflammation and is associated with several systemic diseases, such as diabetes, cardiovascular disease, and rheumatoid arthritis. In fact, the relationship can be reciprocal, as in the case of diabetes, where periodontitis and diabetes negatively impact each other. This can be due to the release of inflammatory cytokines during chronic periodontitis, which results in a sustained systemic inflammatory response [77]. Evidence shows that NLRP3 plays a role in glucose tolerance, insulin resistance, and inflammation in both type 1 and type 2 diabetes. In patients with poorly controlled type 2 diabetes, those with chronic periodontitis displayed higher *caspase-1* mRNA levels and elevated protein levels of NLRP3, ASC, and IL-1β compared with periodontitis patients who were not diabetic [78]. Additionally, a relatively recent report confirmed the increased levels of NLRP3 in the saliva or serum of patients with moderate periodontitis than healthy individuals or those with diabetes only [79].

The increase in the levels of NLRP3 inflammasome components may indicate their involvement in the exaggerated periodontal inflammation as well as in the pathogenesis of diabetes. In agreement, an in vitro study reported that gingival fibroblasts expressed an increase in NLRP3 expression upon stimulation with glucose and LPS; however, inhibition of NLRP3 induced a significant decrease in caspase-1 and Il-1β expression levels. Therefore, in a hyper-glucose state, the NLRP3 inflammasome may be involved in an exaggerated innate immune response that further exacerbates gingival inflammation [80].

In addition to the role of NLRP3, AIM2 has been suggested to play a role in the pathogenesis of type 2 diabetes (T2D) as its expression levels were reported to be upregulated in patients with T2D compared to healthy controls. This may be due to high levels of circulating cell-free mitochondrial DNA in T2D that may engage with AIM2, initiating oligomerisation and release of IL-18 and IL-1β [81]. Hence, contributes to the inflammatory process in T2D. Indeed, a recent study reported the association between AIM2 and IFI16 salivary levels and periodontal status in patients with T2D. Interestingly, a significant positive correlation existed between clinical periodontal parameters in type 2 diabetic patients, such as the gingival index, clinical attachment loss, periodontal inflamed surface area and salivary levels of AIM2, IFI16, and IL18 [82].

In rheumatoid arthritis (RA), the prevalence of periodontitis and clinical attachment loss is much higher than that in healthy individuals [83]. Remarkably, the severity of RA was found to correlate with the severity of periodontitis [84]. Strong evidence suggests the involvement of the NLRP3 inflammasome in the pathogenesis of RA [85]. As NLRP3 has been reported to be overexpressed in the immune cells of patients with RA [86]. In fact, byproducts of inflammasome activation, IL-1β and IL-18, are induced by the biomarkers of RA: TNF-α and IL-6. These factors ultimately contribute to activating NLRP3 [86,87]. Additionally, the oral microbiota composition in RA patients is altered, impacting mostly Gram-negative and anaerobic bacteria [88,89]. *P. gingivalis* contributes to the development of RA by catalysing citrullination via its peptidyl arginine deiminase enzyme, resulting in the production of anti-cyclic citrullinated peptide antibodies, an activator of the NLRP3 inflammasome [86,90].

A significant role for NLRP3 has also been suggested in cardiovascular disease (CVD) due to the increase in circulating cytokines [91]. This may be caused by the presence of periodontal and endodontic pathogens, such as *P. gingivalis*, in the circulation along with their PAMPs, directly affecting immune cells [92,93].

**Figure 3:**
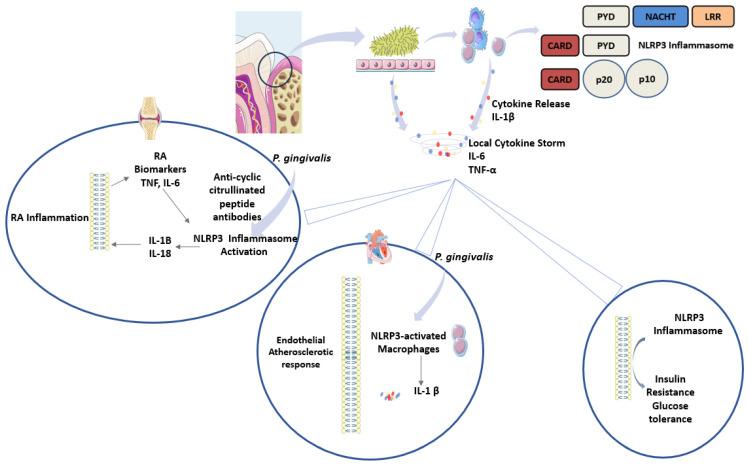
Role of periodontal pathogens in systemic inflammatory disease. Pathogenic bacteria associated with periodontitis induce the release of cytokines from epithelial cells and recruited immune cells. Cytokines prominently involved include IL-1β, IL-6, and TNF-α. This causes a local cytokine storm that can be further amplified by the NLRP3 inflammasome, as evident in rheumatoid arthritis, type 2 diabetes and cardiovascular disease.

Therefore, periodontal pathogens exhibit systemic effects and amplify the inflammatory responses via inflammasome activation in DM, RA, and CVD. Figure 3 summarises the role of periodontal pathogens in amplifying the inflammatory response via inflammasomes and the subsequent systemic effect.

## 5. Chronic Inflammation and Oral Cancer: The Role of Inflammasomes

Head and neck cancer (HNC) is the sixth most common type of cancer accounting for new cases worldwide [94]. As a group of cancers occurring in multiple locations in the head and neck region, including oral cancer (OC), epidemiological trends vary from one region of the world to another; however, its incidence remains high and warrants an epidemic of the disease [95,96]. Unfortunately, patients with oral cancer only have a five-year survival [97]. This is due to late detection, ineffective treatment options, the association of this cancer with eating difficulties, speech impairment and the unavailability of effective treatment options. Since 1863, Virchow noted an association between cancer and inflammation [98]. One of the earliest studies demonstrated an augmentation of cancer growth when injected into inflamed tissue sites [99]. Further evidence showed an active involvement of chronic inflammation in the transition from normal tissue to carcinoma in situ [100]. As described by O’Byrne, prior to the development of most tumours, a pathological state associated with chronic inflammation occurs [4]. However, it was only in the last decade that inflammation was confirmed to play a role in tumorigenesis [101]. Many epidemiological studies identified chronic infection and inflammation as major risk factors for the development of cancers, as (15–20)% of cancer deaths were linked to underlying infections and inflammation [102].

Chronic inflammation creates a cancer-favourable environment due to the promotion of cell proliferation and mutations [103]. Cancer development involves two essential steps: the initiation step, which involves somatic changes that cause permanent DNA damage, induced by viral or chemical carcinogens. The promotion step, where cancer-promoting factors drive excessive cell proliferation, generate reactive oxygen species (ROS), cause oxidative DNA damage, reduce DNA repair capacity, and recruit inflammatory cells through the activation of several PRRs. Particularly, nucleic acid PRRs and inflammasomes such as AIM2, IFI16, cGAS, and STING become activated upon DNA damage or breakage, triggering downstream inflammatory signalling pathways [104,105,106].

Triggers of chronic inflammation that induce cancer arise following exposure to infectious (bacterial or viral) or non-infectious irritants such as chemicals or host-immune abnormalities. The latter may include genetic polymorphisms that increase susceptibility to cancer by enhancing the release of IL-1β and IL-18 [107,108]. These triggers are described next.

### 5.1. Chemical Irritants

Persistent exposure to smoking, betel nut, and alcohol leads to dysplastic alterations that can evolve into malignant transformation [109,110]. Mechanistic studies show that arecoline (alkaloid found in the areca nut) and 4-NQO (a tobacco-related carcinogen) promote IL-1β-driven inflammation through inflammasome activation, both in vivo and in vitro [111]. Tobacco smoking further amplifies pro-inflammatory events in oral epithelial cells and induces vascular endothelial dysfunction by increasing oxidative stress and reactive oxygen species (ROS), a potent trigger of NLRP3 activation [112,113]. Although chronic oral trauma was historically considered a contributing risk factor for oral cancer [114], a recent systematic review reported that chronic physical irritants do not have an active role in oral cancer development [115].

### 5.2. Oral Pathogens

The abundance of commensal flora within the oral cavity mandated an approach to investigate their role in oral carcinogenesis. It was demonstrated that microbial signatures appear to exist in HNC lesions, and perturbation of the normal microflora-host immunity balance potentially plays a role in HNC development [116]. The role of the oral microbiome and the established association of *Helicobacter pylori* (*H. pylori*) with gastric cancer has re-elicited the speculation about a potential relationship between oral commensal microorganisms and oral cancer [117]. Chronic infection with *H. pylori* induces chronic gastritis, which could develop into intestinal metaplasia and eventually into gastric cancer [118]. Interestingly, NLRP3 has been implicated in the development of gastric cancer via IL-1β, as it inhibits acid secretion by gastric cells, enabling *H. pylori* to colonise the gastric epithelium [119].

Periodontal pathogens have been postulated to be involved in the development and progression of cellular carcinoma via varying mechanisms. Interactions between periodontal pathogens and cellular signalling pathways leading to an anti-apoptotic effect were demonstrated. *P. gingivalis* induced significant alterations in the PI3K/Akt and JAK/STAT pathways, resulting in an anti-apoptotic effect [120]. *Treponema denticola* (*T. denticola*) exhibited similar effects via activation of intracellular TGF-β signalling [121]. Furthermore, *Prevotella intermedia* (*P. intermedia*) was demonstrated to induce the release of TNF-α, which is known to promote cell proliferation and oncogenesis via activation of MAPK/ERK and NF-KB [122,123]. Additionally, the AKT2 kinase, regulator of cell survival, proliferation, metabolism and migration, was reported to be upregulated by *Prevotella melaninogenica* (*P. melaninogenica*) in oral potentially malignant lesions. While *Streptococcus mitis* (*S. mitis*) in OSCC lesions correlated with reduced *DUSP16* expression, a deactivator of MAPK. This indicates bacteria-specific effects on host signalling pathways. These findings suggest that microbial dysbiosis may influence molecular changes involved in lesion progression [124]. The activation of such signalling pathways is known to be a prerequisite for inflammasome activation and IL-1β release. Indeed, *P. intermedia* was reported to have a strong correlation with IL-1β levels in periodontal disease [125,126]. *A. actinomycetemcomitans* also promoted the secretion of inflammatory mediators via MAPK, a signalling pathway implicated in IL-1β release [127,128]. Therefore, some virulent periodontal pathogens could activate cellular signalling pathways that control immune responses, IL-1β release and cell life events.

Beyond triggering inflammasome activation, periodontal pathogens also release bacterial products, PAMPs, that can alter cellular oncogenes, ultimately driving abnormal cell proliferation. For instance, *P. intermedia* LPS were reported to stimulate nitric oxide (NO) production and upregulate the RAS oncogenic pathway [129]. Infection with *T. denticola* demonstrated significant epigenetic alterations, which resulted in the production of MMPs, known to break down the extracellular matrix and promote metastasis [130]. *P. gingivalis* potentially promoted tumour progression in oral squamous cell carcinoma (OSCC) cells by modulating T-cell response and enhancing immune evasion of tumour cells [131]. This finding is in agreement with evidence identifying *P. gingivalis* as a prominent periodontitis-related organism with a strong connection to OSCC [132].

All together, this suggests that the release of PAMPs and cellular toxic substances with the subsequent chronic inflammatory responses that follow could inflict genetic and epigenetic alterations, thus providing a sustainable inflammatory environment conducive to the development and progression of cancer.

In addition to initiating inflammation and driving genetic and epigenetic changes that promote cancer, oral pathogens continue to play an active role within the OSCC tumour environment itself. Studies have reported the activation of NLRP3 in cells of OSCC by PAMPs such as LPS with ATP [133]. Certainly, *P. gingivalis* and *F. nucleatum* promoted the proliferation and progression of OSCC cells in vitro by overexpressing *NLRP3* and activating the ATR-CHK1 molecules, promoting tumour growth. In mouse models, *P. gingivalis* and *F. nucleatum* exacerbated bone resorption, increasing the tumour mass [134]. Additionally, *F. nucleatum* further promoted tongue squamous cell carcinoma progression by inducing dsDNA breaks and altering the Ku70/p53 DNA reparative pathways in OSCC cell lines [135]. Furthermore, *P. gingivalis* was capable of activating and upregulating NLRP3 and AIM2 alone or in combination with *F. nucleatum* in epithelial-derived cells [136]. Therefore, these pathogens can accelerate the pathogenesis of HNC by releasing toxins, disrupting inflammasome regulation, sustaining chronic inflammation, and facilitating immune evasion—collectively creating a pro-tumorigenic environment. Although these studies demonstrated that periodontal pathogens can modulate NLRP3 expression in vitro and in vivo mouse models, they did not explore the role of AIM2 in tumour growth in vivo or examine AIM2 activation in response to bacteria-induced DNA damage. In addition, none of these models employed inflammasome inhibitors. Future studies are therefore needed to test pharmacological blockers or inhibitors of inflammasomes to determine whether they can reverse or limit the harmful consequences of inflammasome activation. Importantly, such work should also address key unresolved questions: Would inhibiting inflammasomes make bacteria more virulent or, conversely, reduce their pathogenic impact? Would tumour progression slow down or potentially worsen? In other words, do inflammasomes exert a predominantly protective role in this context, a detrimental one, or both, depending on disease stage and microenvironment?

### 5.3. Oncogenic Viruses

Many viral agents have been associated with cancer development. Human papillomavirus (HPV), the causal factor for cervical cancer, has been reportedly linked to HNC, including OSCC. HPV subtypes 16 and 18 have been linked to oropharyngeal cancer, with subtype 16 being the most commonly associated with its development [137,138]. The activation of NLRP3 inflammasome is important in the pathogenesis of HSV subtype 1, which is associated with oral and genital lesions [139]. However, studies are needed to confirm the association between NLRP3 and herpes simplex virus (HSV) subtypes that induce oral cancer. Interestingly, HSV-1 inhibited AIM2 activation via its tegument protein, a tactic to evade immune responses and enable viral replication [140].

Recent evidence suggests that EBV may also upregulate Notch-signalling components in oral lesions, potentially contributing to local bone resorption and inflammatory pathology [141,142]. Additionally, long-standing infection with Epstein–Barr virus (EBV) induced Burkitt’s lymphoma and nasopharyngeal cancer (NPC) [143]. In fact, AIM2, NLRP3 and RIG-1 were overexpressed in EBV-associated NPC and were interestingly reported to possess anti-tumour effects in EBV-associated NPC via IL-1β release [144].

Additionally, Human Herpes virus 8 (HHV8), which is involved in the development of Kaposi sarcoma, inhibited inflammasome activation, thus suppressing anti-viral immunity and facilitating a sustained viral infection state [145]. Therefore, a differential role for inflammasomes in tumour development and progression exists; they can be pro-tumorigenic or anti-tumorigenic [146].

### 5.4. Immune-Mediated

Chronic inflammation plays a role in the pathogenesis of potentially malignant lesions such as oral leukoplakia (OL), oral erythroplakia (OE) and oral lichen planus (OLP) [147]. The World Health Organisation (WHO) considers OLP as a pre-malignant condition due to the rate of patients who develop oral squamous cell carcinoma [148]. It is a chronic immune-inflammatory T cell-mediated disease with many cytokines contributing to its development and persistence, such as TGF-β1, IL-12, IL-4, IL-10, IL-6, GM-CSF, and IL-1β [149]. Mainly, IL-6 and IL-1β are produced by infiltrated mononuclear cells, stimulating the release of TNF and angiogenic factors such as VEGF, which are implicated in tumour initiation and progression [150]. O-GlcNAcylation regulates diverse cellular processes and is essential for keratinocyte differentiation and cell adhesion [151]. O-linked β-N-acetylglucosamine proteins have been detected in OLP and are well-known promoters of NFKb [152]. Interestingly, NLRP3 has been suggested to play a role in OLP, as a study reported increased levels of NLRP3 inflammasome components in OLP lesions compared to healthy lesions. Evidence suggests this amplification is related to an increase in O-linked β-N-acetylglucosamine proteins detected in OLP, which are promoters of NFKb, as previously mentioned; they may ultimately induce the expression of NLRP3 inflammasome components [152]. In addition, heat shock proteins were demonstrated to have an altered expression in OLP lesions [153]. HSPs are post-transcriptional regulating proteins and well-known DAMPs and activators of inflammasomes [154]. HSPs have been speculated to be involved in OLP pathogenesis via NLRP3. All in all, NLRP3 potentially plays a role in the pathogenesis of OLP. However, to this day, the causative antigen of OLP remains unknown, and further studies are needed to identify it.

In Sjögren syndrome (SS), a chronic inflammatory disorder, aberrant activation of the NLRP3 and AIM2 inflammasome pathway was confirmed. Causes of such activation occurred as a result of the deposition of extracellular DNA and excessive buildup of cytoplasmic DNA in the epithelium of salivary ducts, respectively [155]. Interestingly, IFI16 is also activated in the ductal epithelia due to the accumulation of cytoplasmic dsDNA [156]. Thus, NLRP3, AIM2 and IFI16 are components of the innate immune system that are activated in Sjögren syndrome. These inflammasomes contribute to the pathogenesis of this disorder.

Patients with Sjogren syndrome are more susceptible to developing cancers such as non-Hodgkin B-cell lymphoma, among other cancers, and oral cancer. One case has been reported about the co-existence of SS with nasopharyngeal carcinoma [157]. However, whether inflammasomes play a role in this needs further elucidation.

**Figure 4:**
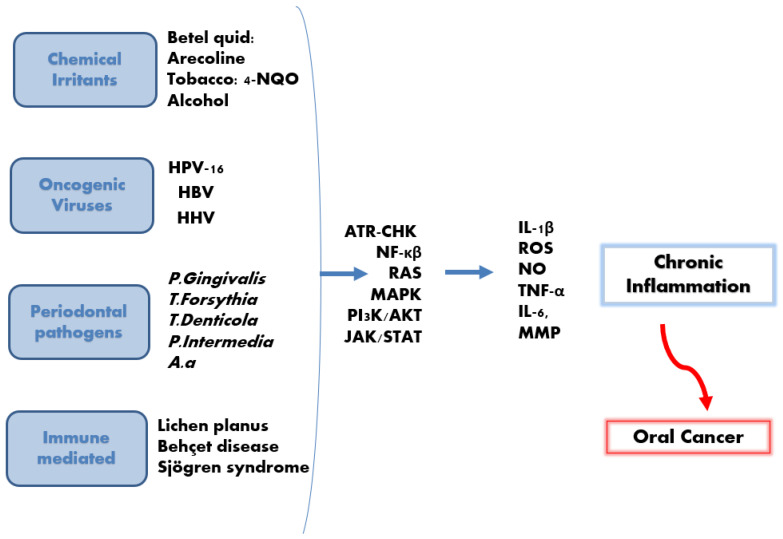
Chronic inflammation and oral cancer development. Chemical, bacterial, viral, and immune-mediated diseases induce the activation of many signalling pathways, resulting in the release of many mediators, including IL-1β. This is followed by subsequent chronic inflammation, which may potentially develop into oral cancer.

Another inflammatory disorder related to inflammasomes is Behçet disease (BD). BD is an inflammatory multi-systemic disease affecting blood vessels and tissues, which causes painful oral and genital ulcers. Patients suffering from BD suffer from elevated serum levels of IL-18 and IL-1β. NLRP3 inflammasome has been implicated in the pathogenesis of BD [158], and it is also possible that AIM2 plays a role; however, further studies are needed to confirm that. Interestingly, patients with variants in the IFI16 gene patients with 2 single nucleotide polymorphisms (rs6940) in IFI16, were reported to be at a higher risk for developing BD [32]. Figure 4 summarises the various types of cancer promoters. Table 1 illustrates the vital inflammatory mediators and biomarkers of chronic pulpitis, chronic periodontitis and oral cancer.

## 6. The Role of Inflammasomes (NLRP3, AIM2, IFI16), Nuclear Factor Kappa B (NFκB), Interleukin-1 Beta (IL-1β), in the Pathogenesis of Oral Cancer and Potential Therapeutic Targets

Biopsied tissues from patients with OSCC confirmed an upregulation in NLRP3 and its inflammasome components (ASC, IL-1β and caspase-1). Additionally, IL-1β, CASP1, and NLRP3 were all strongly linked to key clinical features of OSCC, with ASC showing the most consistent association across all the clinicopathological characteristics examined. ASC levels also positively correlated with tumour stage and poor prognosis, making ASC a potential prognostic marker [110]. Similarly, an upregulation of all NLRP3 inflammasome components was observed in tissue samples taken from patients with head and neck cancer. In fact, an increase in the tendency of cancer recurrence and metastasis was observed with increased mRNA expression levels of NLRP3, ASC, IL-1β, and caspase-1. Notably, the purinergic P2 × 7 receptor, responsible for providing the ATP-dependent second signal required for NLRP3 activation, was also markedly elevated [175]. Importantly, inhibiting either P2 × 7 or NLRP3 reduced the invasive behaviour of OSCC cells, and this effect was even more pronounced when both pathways were blocked simultaneously using oATP and MCC950, the respective inhibitors of P2 × 7 and NLRP3 [175]. In a parallel study, the expression of NLRP3 inflammasome components correlated with the expression of cancer stem cell markers [133]. However, upon utilising MCC950, a delay in tumorigenesis in vivo mouse model was confirmed, as well as downregulation in the cancer stem cell phenotype [133]. Another study with a similar focus reported the correlation of NLRP3 levels with OSCC tumour progression, tumour stage, lymph node involvement and metastasis. Knocking down NLRP3 in OSCC cells reduced OSCC proliferation, migration, and invasion considerably in vitro and in vivo mouse model [6]. In accordance, upon using an NLRP3 inhibitor, BAY117082, the NLRP3 inflammasome components expression and activation were inhibited, resulting in the reduction in OSCC cell viability in vitro and a reduction in tumour mass in vivo mouse models [176]. Collectively, this evidence confirms a role for NLRP3 inflammasome and its components in OSCC progression and metastasis; however, these studies relied mostly on cell line models, which can not fully replicate the complex tumour environment. The mouse models used did not examine long-term tumour progression. Therefore, well-designed longitudinal studies in human cohorts are needed to clarify the true clinical impact of inflammasome activation in OSCC.

In addition to NLRP3 being implicated in chronic inflammation and cancer progression, the dsDNA-sensing PRRs, AIM2 and IFI16, have been involved in oral diseases and cancer [27].

During carcinogenesis and upon cell stress, the nuclear membrane is damaged, resulting in the release of host DNA into the cytoplasm and its recognition by AIM2 propagating inflammation [177,178]. In OSCC samples of smoking patients, AIM2 expression was upregulated in comparison to normal non-smoking samples [179]. AIM2 was confirmed to contribute to OSCC progression by inducing cell migration, invasion, and epithelial–mesenchymal transition [180,181]. However, in colorectal cancer and prostate cancer, AIM2 acted as a tumour suppressor by supporting genomic stability via regulating DNA-PK (repairs breaks in dsDNA) and PI3K/Akt pathways (promotes proliferation and survival) [182,183]. Therefore, AIM2 can act as a tumour promoter or tumour suppressor due to differences in tissue-specific signalling, the nature of DNA damage, and variations in the tumour microenvironment.

Chemotherapeutic agents or cytotoxic drugs used in cancer treatment activate AIM2 by inducing tumour cell death and release of endogenous DAMPs such as ATP, HMGB, and self-dsDNA [178]. Intestinal Cells and bone marrow cells exposed to ionising radiation or chemotherapeutic agents (etoposide and doxorubicin) undergo dsDNA breakage and leakage of self-DNA, inducing inflammatory responses and cell death mediated by AIM2 inflammasome. However, AIM2-deficient cells and mice are protected from cell death and subtotal body irradiation-induced lethality. This suggests that AIM2 could be used as a therapeutic target to minimise the detrimental inflammatory output following radiation [177,178]. In addition, NLRP3 was confirmed to play a role in the chemoresistance of OSCC cells to the 5-Fluorouracil chemotherapeutic drug in vitro and in vivo hence targeting the NLRP3 inflammasome pathway may enhance the 5-Fluorouracil chemotherapy of OSCC [8]. However, further work is needed to unravel the specific mechanisms through which these proteins operate in diverse tumour settings and to assess their value as potential therapeutic targets in chemotherapy or future cancer therapies.

IFI16 has been implicated in the development of OSCCs, as high expression levels of IF16 and AIM2 were reported in OSCC samples following a high-density single-nucleotide polymorphism (SNP) array study [7]. Additionally, overexpressing IFI16 alongside AIM2 was reported to play pro-tumorigenic roles in OSCC, via promoting cell growth and inhibiting apoptosis through NFκB activation [180].

NFκB is a key transcription factor that plays an integral role in innate immunity and cellular proliferation. it is upregulated in acute, chronic and auto-inflammatory conditions [184]. Several cytokines activate NFκB, especially TNF-α, IL-1, and IL-18, in a positive feedback loop, and several oncogenes influence NFκB. The detection of PAMPs and/or DAMPs by PRRs induces the downstream activation of NFκB. In addition, inflammasome activation also culminates in the NFκB activation and transcription of NLRP3 inflammasome components alongside its products IL-18, IL-1β [185]. It has long been postulated that NFκB promotes oncogenesis as it is an activator of anti-apoptotic genes, making it an important tumour-promoting factor [186]. In colitis-associated cancer, the ikkb-NFκB signalling pathway had a pro-tumorigenic role in enterocytes and myeloid cells. As in the enterocytes, anti-apoptotic genes were activated via ikkb-NFκB, and in myeloid cells, growth factors of pre-malignant cells, IL-6 and IL-8, were produced via ikkb-NFκB [187]. In addition, NFκB activation ensures the survival, progression and obstruction of tumour surveillance mechanisms [188].

In squamous cell carcinoma cells of HNC, NFκB was highly expressed, and its level of expression correlated with the increased invasiveness and metastasis in these cells [189]. Indeed, NFκB was confirmed to be basally upregulated along with genes encoding for interleukins, chemokines, proliferation and angiogenesis [190]. Additionally, the role of NFκB in promoting the invasion of OCSS was confirmed [188]. The p65, the main component of NFκB, was upregulated in OSCC invasive cells, tumour-associated stroma cells and osteoclasts. Furthermore, NFκB was essential for osteoclast functions, as inhibiting NFκB reduced the mandibular bone invasion and proliferation of tumour cells without affecting normal host cells [188]. Interestingly, the administration of anti-inflammatory drugs, aspirin and NFκB inhibitors, reduced the survival ability of OSCC. Thus, modulating NFκB may be a potential target for OSCC therapy in OSCC cell lines. Additionally, some studies revealed that suppression of NFκB in cancer cells was associated with enhanced sensitivity to chemotherapeutic agents [191].

To further elucidate the role of inflammasomes in cancer, a closer look at their by-product IL-1β is needed. IL-1β, a key mediator of inflammation, as it induces the expression and release of many pro-inflammatory cytokines by stromal or inflammatory cells, resulting in the propagation and maintenance of an inflammatory environment [192]. This critical cytokine is tightly controlled via inflammasomes, as explained previously [193].

Importantly, this same inflammatory potential also links IL-1β to cancer progression. For example, IL-1 and TNF encourage the adhesion of tumour cells to endothelial cells in a dose and time-dependent manner [194]. Likewise, IL-1β was confirmed to play a role in the metastasis of different tumour types to the lung, such as human colon carcinoma, human melanoma and renal carcinoma [195]. Consistently, IL-1β has been reported to have a pro-tumorigenic role in various cancers, such as lung cancer, GI cancer, melanoma and HNC [196,197]. Although not an oncoprotein itself, IL-1β promotes the release of oncogenic cytokines such as IL-6 and IL-8 from cancer cells.

In biopsied tissues of OSCC and leukoplakia, IL-1β levels rose progressively as tissues advanced through the stages of malignant transformation. Upon silencing IL-1β, OSCC progression was inhibited in both in vivo models and in vitro [196]. In opposition to earlier reports, a recent study confirmed a decrease in the expression of IL-1β and IL-8 in OSCC compared to OLP and leukoplakia. According to the authors, multiple factors may account for this observation; the normal mucosa samples were taken from periodontal tissue, which may explain the persistent expression of both cytokines even under clinically healthy or minimally inflamed conditions. A loss of keratinocyte inflammasome activity in malignant cells was also suggested, which might explain the lower IL-1β levels and may suggest a reduction in pyroptosis. The decrease in IL-8 can be related to the decrease in IL-1β, as one of the cofactors that stimulates its production [198].

Interestingly, a newly published study identified histone deacetylase-6 (HDAC6) as a promising therapeutic target, showing that its inhibition can reduce inflammation and alter the tumour microenvironment in a way that limits OSCC progression. HDAC6 plays a major role in driving OSCC by favouring a pro-inflammatory environment by enhancing IL-1β production, partly through its deacetylation of α-tubulin, which supports the movement and secretion of IL-1β–containing vesicles. Using both in vivo mouse models and in vitro cell studies, HDAC6 and IL-1β progressively increased as OSCC advanced, a pattern also reflected in human cancer datasets (TCGA-HNSCC). Notably, inhibition of HDAC6 with Tubastatin A markedly slowed tumour growth, lowered IL-1β levels, and shifted the immune landscape toward an anti-tumour profile; reducing M2 macrophages while enhancing M1 macrophage responses. Mechanistically, TSA achieved this by blocking the microtubule-dependent pathway required for IL-1β secretion [199]. Although TSA maybe a promising approach, validation in human OSCC tissue is also needed, correlating the expression of IL-1 and HDAC6 with survival, staging, and lymph node status.

Similarly to its established role as a diagnostic biomarker in periodontitis, emerging evidence indicates that IL-1β may also support the early detection and prognostic evaluation of OSCC. Notably, IL-1β levels in unstimulated saliva were found to be significantly higher pre-operatively compared with post-operative levels in patients with OSCC [200]. This reinforces the pro-tumorigenic influence of this cytokine in oral carcinogenesis. Consistently, a recent systematic review reported elevated salivary concentrations of IL-1β, IL-1α, IL-8, IL-6, and TNF-α in patients with oral cancer when compared with healthy controls [165].

### Limitations

This review is narrative in nature, which comes with certain methodological limitations. The search was not conducted using a predefined protocol and was limited to English-language studies available in major scientific databases; thus, some relevant work may not have been captured. Because no formal risk-of-bias assessment or structured quality appraisal was performed, the strength of the conclusions is influenced by the varying quality and heterogeneity of the studies included. Additionally, the evidence base covers a wide range of experimental models, cell culture, animal studies, observational clinical studies, and small patient cohorts, which may limit direct comparability and prevent definitive causal inference. Accordingly, these findings should be viewed as a summary of the main patterns emerging in the literature, rather than a formal assessment of how strong or conclusive the evidence is.

## 7. Conclusions

Overall, this review synthesises current scientific evidence on the involvement of inflammasomes and IL-1β in chronic inflammatory oral conditions (primary outcome) and explores how these inflammatory pathways may contribute to oral carcinogenesis (secondary outcome). Inflammasomes act as key sensors of microbial and damage-associated signals within the innate immune system, and their dysregulated activation has been reported in chronic pulpitis, chronic periodontitis, systemic diseases linked to periodontal inflammation, and oral cancer. IL-1β, a potent multifunctional cytokine regulated by inflammasome activity, is tightly controlled under physiological conditions; however, disturbances in its regulation may contribute to sustained inflammatory responses that can impair tissue repair and disrupt homeostasis. While the causes of oral cancer are multifactorial, chronic inflammation is a common feature across many established risk factors, and inflamed sites appear more susceptible to malignant transformation than non-inflamed tissues. Although most available studies describe associations rather than definitive causal pathways, investigating the regulatory mechanisms governing inflammasome activation and IL-1β signalling remains essential. Such work may help clarify how persistent inflammation influences disease progression and could ultimately inform strategies aimed at mitigating harmful inflammatory responses and supporting tissue homeostasis.

## Figures and Tables

**Table 1:** The key inflammatory mediators across chronic pulpitis, periodontitis and oral cancer.

Inflammatory Mediator	Chronic Pulpitis	Chronic Periodontitis	Oral Cancer
Interleukin-6 (IL-6)	Biomarker of chronic pulpitis [159].	Proinflammatory in periodontitis, putative role in systemic sequel in periodontitis [160].	Elevated in the saliva of patients diagnosed with OSCC [161]. Promotes tumour growth, angiogenesis [162]
Interleukin-8 (IL-8)	Neutrophil chemoattractant and biomarker of chronic pulpitis [163].	Induction of other inflammatory mediators. Increased in GCF in periodontitis [164]	Strong salivary biomarker; associated with poorer survival [165]
Tumour necrosis factor-alpha (TNF-α)	An early-stage mediator coordinates inflammatory responses in deeply carious dental pulps and deeper associated periapical infections [166].	Key and early-stage mediator [63]. Activation of osteoclast in vitro [164].	Elevated in saliva; TNF-α polymorphisms increase OSCC risk. NF-κB activation; promotes invasion [165,167]
Interleukin-18 (IL-18)	Pro-inflammatory mediator and increased in inflamed pulps [168].	Significant increase in saliva, serum, and GCF in periodontitis, suggested as biomarker for periodontitis [169].	Elevated levels of serum IL-18, a systemic biomarker for OSCC [170].
Interleukin-1 beta (IL-1β)	The master regulator of cytokines; induces their expression of cytokines such as IL-8 [171].	A key mediator of inflammation and early-stage mediator [63]. Increase MMP expression and subsequent bone resorption [58].	Elevated in saliva of patients diagnosed with OSCC; a prognostic biomarker [165].
Matrix metalloproteinase (MMP)	MMP-1, MMP-8, MMP9, and MMP-13 are biomarkers of chronic pulpitis [171].	MMP-8, MMP-13 have increased levels in chronic periodontitis [58,164]	MMP-9 activated by *P. gingivalis* inducing cancer invasion and metastasis [172]. Increased levels in saliva of patients with OSCC [173].
Interleukin-10 (IL-10)	Anti-inflammatory; inhibits the release of pro-inflammatory cytokines [171]	Anti-inflammatory effect, suppression of osteoclastic activity [59]	Elevated in saliva/tissue; poor prognosis marker [174].

## Data Availability

Not applicable.
